# Membrane Environment Modulates Ligand-Binding Propensity of P2Y12 Receptor

**DOI:** 10.3390/pharmaceutics13040524

**Published:** 2021-04-09

**Authors:** Fatemeh Haghighi, Semen Yesylevskyy, Siamak Davani, Christophe Ramseyer

**Affiliations:** 1 Laboratoire Chrono Environnement UMR CNRS 6249, Université de Bourgogne Franche-Comté, 16 route de Gray, CEDEX, 25030 Besançon, France; fateme.haghighi@hotmail.com (F.H.); yesint4@gmail.com (S.Y.); 2 Unité de Recherche EA 3920, Université de Bourgogne Franche-Comté, 19 rue Ambroise Paré, CEDEX, 25000 Besançon, France; siamak.davani@univ-fcomte.fr; 3 Department of Physics of Biological Systems, Institute of Physics of the National Academy of Sciences of Ukraine, Prospect Nauky 46, 03028 Kyiv, Ukraine; 4 Laboratoire de Pharmacologie Clinique et Toxicologie-CHU de Besançon, 3 boulevard A. Fleming, CEDEX, 25030 Besançon, France

**Keywords:** ticagrelor, P2Y_12_ receptors, platelets, molecular dynamics

## Abstract

The binding of natural ligands and synthetic drugs to the P2Y12 receptor is of great interest because of its crucial role in platelets activation and the therapy of arterial thrombosis. Up to now, all computational studies of P2Y12 concentrated on the available crystal structures, while the role of intrinsic protein dynamics and the membrane environment in the functioning of P2Y12 was not clear. In this work, we performed all-atom molecular dynamics simulations of the full-length P2Y_12_ receptor in three different membrane environments and in two possible conformations derived from available crystal structures. The binding of ticagrelor, its two major metabolites, adenosine diphosphate (ADP) and 2-Methylthioadenosine diphosphate (2MeS-ADP) as agonist, and ethyl 6-[4-(benzylsulfonylcarbamoyl)piperidin-1-yl]-5-cyano-2-methylpyridine-3-carboxylate (AZD1283)as antagonist were assessed systematically by means of ensemble docking. It is shown that the binding of all ligands becomes systematically stronger with the increase of the membrane rigidity. Binding of all ligands to the agonist-bound-like conformations is systematically stronger in comparison to antagonist-bound-likes ones. This is dramatically opposite to the results obtained for static crystal structures. Our results show that accounting for internal protein dynamics, strongly modulated by its lipid environment, is crucial for correct assessment of the ligand binding to P2Y12.

## 1. Introduction

Platelets are major drug targets in case of thromboembolic disorders. Pharmacological inhibition of platelet aggregation is necessary for preventing complications after an acute coronary syndrome (ACS) and the formation and progression of thrombotic processes. Adenosine diphosphate (ADP) is an important signal molecule for platelet function, which is involved in their physiological and pathological responses [1]. The inhibition of ADP-induced platelet aggregation is an effective approach for treating thrombotic events in clinical practice. ADP binds to purinergic receptors P2Y1 and P2Y12 which are seven-transmembrane (7TM) G-protein-coupled receptor (GPCR) located in the platelet cell membrane [2]. These receptors can be activated by extracellular nucleotides and trigger the completion of the platelet aggregation [3,4]. Therefore, ADP receptors are nowadays considered as the main targets for antiplatelet agents [5]. The P2Y12 receptor is abundantly expressed in the platelet membrane [6] and is a key player in primary hemostasis and arterial thrombosis, which makes it one of the most prominent drug targets for the inhibition of platelet aggregation [5,6].

Currently, two classes of P2Y12 antagonists have been developed and approved by the FDA as antiplatelet agents, namely the irreversible thienopyridines and the reversible ATP analogues. Thienopyridine compounds such as ticlopidine, clopidogrel, and prasugrel irreversibly inhibit P2Y12 receptor [7]. They are prodrugs that must be metabolized in the liver [8]. The active metabolite of clopidogrel and prasugrel covalently binds to cysteine residues of P2Y12 precluding the binding of ADP [9,10]. Reversible P2Y12 receptor antagonists are modeled after ATP as a scaffold [11]. Ticagrelor is the compound from this group that is widely used in clinical practice [11,12]. It binds to the receptor directly without the need for hepatic bioactivation [13], which results in a faster onset of action, less interindividual variation in effectiveness, and greater control of platelet aggregation inhibition [14]. It is reported that the primary metabolite of ticagrelor is also active with potency equal to the parent compound [12].

Limitations of existing drugs, such as a very long half-life of clopidogrel and adverse effects of ticagrelor [11,15], indicate that there is an unfulfilled demand for a new generation of P2Y12 inhibitors. Unfortunately, ligand recognition and the function of P2Y12 remain poorly understood at the molecular level due to unclear or even conflicting structure–activity relationship (SAR) data [16,17], which hampers the development of new inhibitors. It is well known that the lipids regulate the location and activity of many membrane proteins as well as forming lipid microdomains that control the localization and interactions of proteins involved in cell signaling [18,19]. Being an integral membrane protein, P2Y12 receptor is also likely to be influenced and regulated by its lipid environment. Particularly, the impact of ticagrelor on the lipid composition of platelet plasma membrane [20] and the role of cholesterol on the activation of P2Y12 receptor was recently studied [21]. It was shown that the location of P2Y12 in ordered lipid microdomains (“rafts”) is required during ADP-mediated platelet activation [21]. Moreover, the active metabolite of clopidogrel breaks up the homooligomers of P2Y12 receptor into non-functional dimers and monomers that are located outside of lipid rafts [10]. The effect of ADP and ticagrelor on the detergent-resistant membrane (DRM) was also examined recently [22].

Experimental studies of the influence of lipid environment on membrane proteins are complex, tedious, and time consuming, which recently boosted the development of computational approaches to this problem. In the case of P2Y12, early numerical studies [23,24] were pushed forward by resolution of the crystallographic structures of the P2Y12 complexed with the full agonist 2-methylthioadenosine-5′-diphosphate (2MeSADP, PDB ID: 4PXZ), with the partial agonist 2-methylthio-adenosine-5′-triphosphate (2MeSATP, PDB ID: 4PY0) [25] and with the antagonist ethyl 6-(4-((benzylsulfonyl)carbamoyl)piperidine-1-yl)-5-cyano-2-methylnicotinate (AZD1283, PDB ID: 4NTJ) [25]. These structures differ substantially by the conformation of transmembrane helix 6 (TM6). In the agonist-bound 4PXZ, this helix is bent inwards toward the active site (this structure will be referred as “closed” hereafter). In antagonist-bound 4NTJ, this helix is straight, and the active site is significantly more exposed (this structure will be called “open” hereafter). These large-scale rearrangements in the highly malleable extracellular regions are required to allow agonist accessing the binding pocket. The previous structural and docking studies [26] were not able to provide valuable insight into the pharmacology and mechanisms of action of agonists and different classes of antagonists because they were performed before these structural differences become known. For instance, ticagrelor could not be docked into a pose similar to one of 2MeS-ADP because the presence of bulky N6 substituents would cause a steric clash with the helixes V and VI [26].

Recently, an extensive molecular docking study of the major classes of substances, previously reported as P2Y12 ligands, was performed [27]. The authors tried to rationalize the main SAR findings previously reported for each class of the ligands. It was shown that antagonists such as ureas, sulfonylureas, sulfonamides, anthraquinones, and glutamic acid piperazines docked readily to the antagonist-bound P2Y12 structure, while various nucleotide derivatives docked readily to the agonist-bound structure. However, they were unable to dock ticagrelor to the agonist-bound P2Y12 structure. The “hybrid” receptor resembling the agonist-bound P2Y12 except for the top portion of TM6, which was taken from the antagonist-bound P2Y12 structure, was necessary for successful ticagrelor docking. This example shows the limitations of available P2Y12 models and emphasizes a need for more elaborate computational studies, which take into account protein mobility and conformational changes in its native membrane environment.

In the present study, we propose the most realistic computational model of P2Y12 available to date. First, we consider the receptor in its native lipid bilayer environment. The dynamics of the receptor is investigated as a function of bilayer composition. Phosphatidylcholine (PC), sphingomyelin (SM), and SM/cholesterol mixture (referred as “raft” hereafter) environments are studied. Second, both agonist and antagonist-bound crystal structures are used as initial conformations in each lipid environment. Third, an extensive ensemble docking analysis is made on a representative set of simulation snapshots extracted from equilibrated molecular dynamics (MD) trajectories, which takes into account both the local flexibility of the binding site and the large-scale protein motions. This methodology was already used with great success for the proteins with complex internal dynamics, such as STAT1/STAT3 [28,29] and human serum albumin [30,31].

We demonstrate that the lipid environment imposes significant constraints into the dynamics and internal flexibility of P2Y12, which in turn influences its binding to a variety of ligands. In general, the more ordered the lipid environment, the higher the binding propensity of P2Y12 to all studied ligands. The bulky nucleotide ligands, such as ticagrelor, can access the binding pockets of both agonist and antagonist-bound P2Y12 conformations with the help of internal protein dynamics.

## 2. Materials and Methods

### 2.1. Homology Modeling

In this work, we considered two crystal structures of P2Y12: 4PXZ:A in complex with 2MeS-ADP agonist [26] (referred as “closed” conformation) and 4NTJ:A in complex with AZD1283 antagonist drug [25] (referred as “open” conformation).

The closed structure of P2Y12 (PDB code 4PXZ:A) was chosen as the starting point for homology modeling. It covers only the residues 15–305, which constitute the major part of transmembrane core of this membrane protein. The crystallized structure is an engineered chimeric protein with soluble cytochrome B562 inserted between the residues 223 and 224 of P2Y12. According to UniProt annotation (UniProtKB: Q9H244), the secondary structure of the missing regions 1–14 and 306–342 is not known and presumed to be unstructured.

The homology modeling approach was used to reconstruct the full-length structure of P2Y12. The cytochrome insertion was removed, and the GalaxyTBM server [32] was used for homology modeling based on the full-length sequence. There are no suitable homology templates for the long C-terminal region 306–342; thus, the structure generated for this region was not satisfactory. Particularly, it protruded deep into the membrane, while the C-terminus should be located in the cytoplasm according to UniProt annotation. That is why we performed de novo structure prediction for the region 302–342 using the QUARK server [33]. Then, the predicted structure was aligned with the rest of the protein using the overlapping helical region 302–305 and manually attached to the transmembrane segment.

In order to construct the open structure of the protein, we first aligned the obtained homology model of the closed form with the crystal structure 4NTJ:A and then transferred all atomic coordinates from the crystal structure to our model. This resulted in the models of closed and open conformations which share the same reconstructed peripheral regions missed in the crystal structures.

### 2.2. Binding Pocket Identification

According to the closed crystal structure, the residues reported to bind with 2MES-ADP are ARG19, ARG93, CYS97, SER101, VAL102, TYR105, PHE106, TYR109, MET152, LEU155, SER156, ASN159, THR163, CYS175, LYS179, HIS187, VAL190, ASN191, CYS194, ARG256, TYR259, GLN263, and LYS280 [26]. In the open crystal structure, most of these residues (SER101, VAL102, TYR105, PHE106, TYR109, MET152, LEU155, SER156, ASN159, HIS187, VAL190, ASN191, CYS194, ARG256, TYR259, LYS280) also bind to AZD1283 [25]. Some other residues such as GLN195, PHE252, ALA255, THR260, LEU276, VAL279, and THR283 were also involved in binding [25]. The center of masses of all these residues was used in this work to determine the center of the binding pocket.

### 2.3. The Ligands

We studied ticagrelor (TIC) and its two major metabolites [34]: an active metabolite AR-C124910XX circulating in the blood (referred as “M8” following the naming in [12]) and the metabolite AR-C133913XX found in the urine (referred as “M5” following the naming in [12]). We also studied ADP, 2MeSADP, and the AZD1283 for comparison with ticagrelor and its metabolites.

### 2.4. Molecular Dynamics Simulations

In this work, the open and closed forms of P2Y12 were inserted into three different lipid environments, namely PC (palmitoyl-oleoyl-phosphocholine, POPC 16:0/18:1, SM (PSM, d18:1/16:0 palmitoylsphingomyelin, and 1:1 SM/cholesterol mixture, which mimics cholesterol-enriched microdomains also widely known as “rafts”. Note that the lengths of fatty acid chains (d18:1/16:0) were reported to be the most abundant ones in platelets membranes [22]. The membranes with inserted protein were generated with the CHARMM GUI membrane builder tool [35,36]. Na^+^ and Cl^–^ ions were added corresponding to ionic strength of ≈0.15 mol/L and adjusted to counterbalance the net charge of the protein. All systems were hydrated to approximately 50 water molecules per lipid. The content of the resulting systems is shown in Table 1.

The membrane ordering and fluidity was assessed by computing lateral diffusion coefficients of the lipids in all studied systems. The results are shown in Table S1 (see Supplementary Materials). It is clearly seen that the PC membrane is an order of magnitude more fluid than SM and raft membranes. In turn, the raft membrane is only slightly less fluid than the SM one. The diffusion coefficients were computed using the mean square displacement (RMS) method as implemented in Gromacs [37] version 5.1.2 by manual fitting of the linear part of the RMS curve.

The open form of the protein was built by substituting the transmembrane core of the closed form with the coordinates from 4NTJ:A crystal structure as described above. The open forms were built separately for three lipid environments from the corresponding pre-equilibrated closed forms.

For all systems, the following equilibration protocol was applied. First, all protein atoms were restrained, and the lipids and solvent were equilibrated for 100 ns. Then, the protein backbone was restrained, and the side chains were equilibrated with the rest of the system for another 100 ns. Finally, all restraints were removed, and the production run of at least 200 ns was performed. The equilibration was monitored by the Root Mean Square Deviation (RMSD) of the transmembrane part of the protein (residues 20–300) excluding the termini, which were fluctuating significantly during the simulations. Simulations that did not converge in 200 ns were prolonged until the RMSD stabilizes.

All MD simulations were performed in Gromacs [37] version 5.1.2 in NPT ensemble at the pressure of 1 atm maintained by Parrinello–Rahman barostat [38] with semi-isotropic pressure coupling. The Verlet cutoff scheme was used [39]. Force-switch cut-off of the Van der Waals interactions was used in the region between 1.0 and 1.2 nm. Long-range electrostatics was computed with the Particle-Mesh-Ewald (PME) method [40] with the cut-off of explicit short-range electrostatic interactions at 1.2 nm. Velocity rescale thermostat [41] was used at the temperature of 310K. The CHARMM36 force field [42] was used for all components of the system. An integration step of 2 fs was used in all simulations with the bonds to hydrogen atoms converted to rigid constraints. Analysis was performed by the custom scripts based on the Pteros molecular modeling library [43].

### 2.5. Ensemble Docking Simulations

In order to account for protein conformational flexibility, multiple conformations from MD trajectories were extracted and used in docking simulations. The last 50 ns of MD trajectories were used to extract 834 equally spaced frames. For each frame, the docking of all ligands was performed. The docking volume was centered in the center of mass of the binding site and had dimensions 2 × 2 × 2 nm. Quick Vina 2 [44] docking software was used with default scoring function. The best docking pose was kept in each simulation. The methodology and scripts for ensemble docking used in our previous works [29,30,31] were used. No side chains were treated as flexible, because the possible protein flexibility was already accounted for by means of MD simulations.

## 3. Results and Discussion

### 3.1. Protein Structure and Flexibility

**Figure 1:**
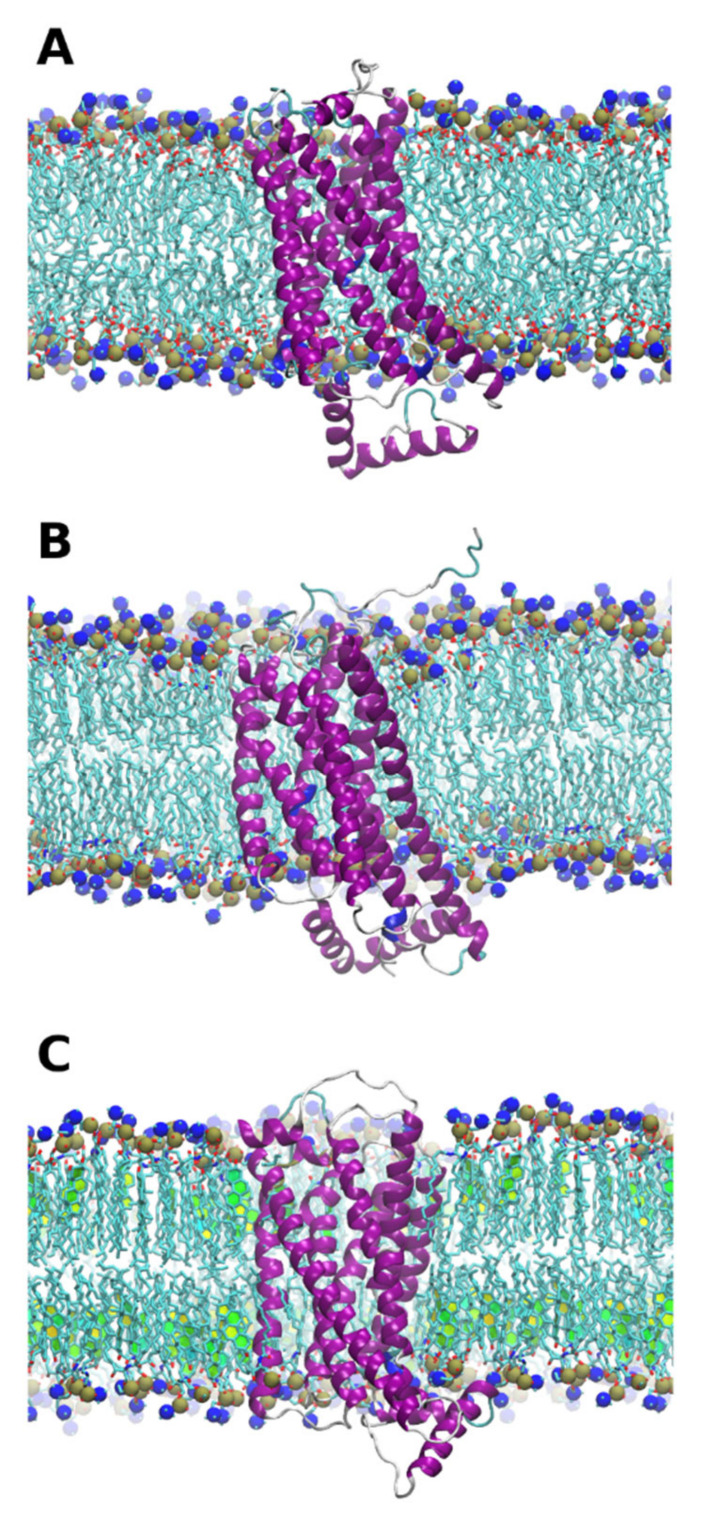
Snapshots of equilibrated closed form of protein in phosphatidylcholine (PC) (**A**), sphingomyelin (SM) (**B**), and SM/Chol (**C**) lipid environments. The protein is shown in cartoon representation. The lipids are shown as sticks with N and P atoms of the head groups rendered as spheres. In (**C**), cholesterol aromatic rings are shown as colored planes. Orientation of the protein is different in different panels. The open form of the protein is hardly distinguishable visually and thus not shown.

Both open and closed forms of the protein were found to be stable in all membrane environments (Figure 1). However, some differences are worth to be noted, as evident from the analysis of RMSD plots (Supplementary Figure S1).

**Figure 2:**
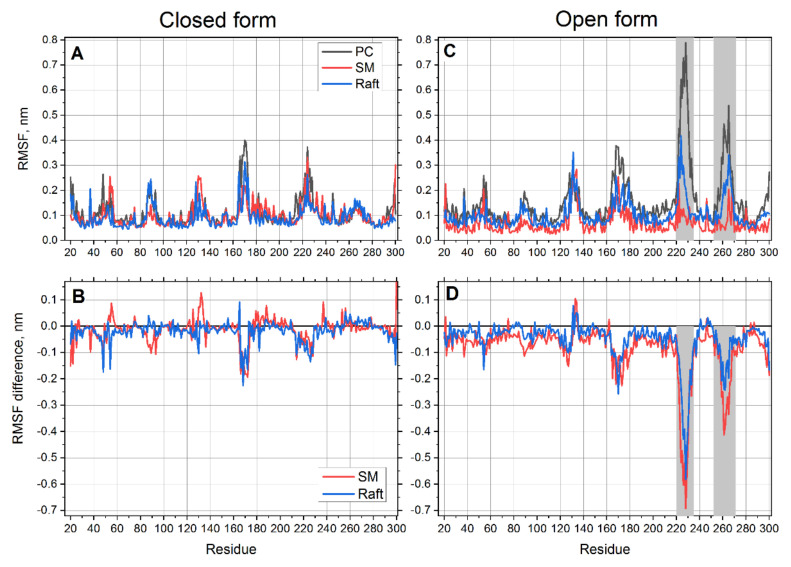
Root means square fluctuations (RMSFs) of the transmembrane region of P2Y12 in different lipid environments for closed (**A**) and open (**C**) forms of the receptor. Differences of RMSFs between SM or raft environments with the reference PC environment for closed (**B**) and open (**D**) forms of the receptor. Filled areas correspond to the regions that are the most sensitive to the change of lipid environment.

First, the equilibration of the protein was significantly longer for the open form than for the closed one. It took up to 400 ns for the open structure in a raft environment. In contrast, the closed form equilibrated in only 200 ns in all three membrane environments. This suggests that the closed crystal structure is closer to equilibrium conformation in a native membrane environment than the open one. Second, the final RMSD for the transmembrane region is less than ≈0.3 nm in the closed form and less than ≈0.5 nm in the open form. These are rather small values for a protein of this size. This means that the structure of the transmembrane part of the protein is in general quite rigid and is not prone to dramatic conformational changes in the course of simulations. The same trend is observed for the active site of the protein, which suggests that the dynamic of the active site is not significantly different from the rest of the protein. The protein termini (residues 1–19 and 301–342), which are exposed to aqueous solution, fluctuate randomly and interact with the membrane transiently. The interaction energies of N and C-terminal regions of the protein with the membranes are shown in Figure S2. The C-terminal domain of P2Y12 binds much stronger to the POPC membrane in both closed and open protein forms in comparison to other membrane compositions. Due to limited sampling, it is not possible to deduce whether this observation is a random coincidence or a systematic effect, but it does not correlate with the overall protein flexibility, behavior of its active site, or the binding propensity of the ligands. Thus, our simulations provide no evidence of a possible direct or indirect influence of the protein termini on the ligand binding and the flexibility of the transmembrane region of P2Y12. Despite overall stability, the protein structure is by no means “frozen” and exhibits enough local conformational mobility as it is evident from the root mean square fluctuations per residue (RMSF), which was computed for the last 50 ns of production trajectories (Figure 2).

The pattern of root mean square fluctuations (RMSFs) in the closed form of the receptor is very similar in all lipid environments (Figure 2A). However, some notable differences could be better visualized on a difference plot, where the data in the PC membrane are used as a reference. The differences of RMSFs in SM and raft membranes from the PC reference are plotted in Figure 2B. It is clearly seen that the RMSFs in both SM and raft membranes are slightly smaller than those in the PC membrane (the values are mostly negative). This trend is more pronounced for the raft mixture. This shows that the closed form is more rigid in the SM and raft environments.

**Figure 3:**
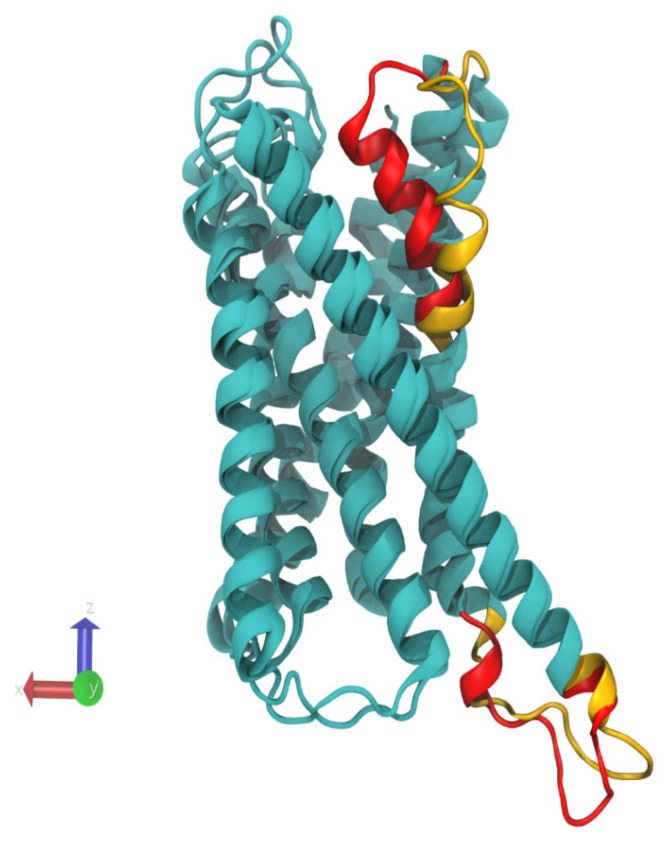
Aligned structures of the closed (PDB code 4PXZ:A) and open (PDB code 4NTJ:A) forms of P2Y12 receptor. Only the transmembrane core (residues 20–300) is shown for clarity. The regions that exhibit the maximal decrease of flexibility in PSM and raft membrane environments are shown in yellow for open form and in red for closed form. The region on top is residues 252–269, and the region on bottom is residues 220–235.

The fluctuations of the open form are more interesting. This structure appears as rigid as the closed one except for two distinct regions (residues 252–269 and 220–235), which are much more flexible than the rest of the protein (Figure 2C). Interestingly, the first region 252–269 corresponds exactly to the transmembrane helix 6 (TM6). This finding corroborates well with the fact that the most noticeable difference between open and closed crystal structures is observed for TM6 [25,26] The other region 220–235 corresponds to the protruding loop on the intracellular side of the protein (Figure 3). Another interesting feature is that flexibility of these two regions in the open form of the receptor varies according to the lipid environment (Figure 2C,D). The protein is found to be the most flexible in PC membrane and significantly less flexible in SM and raft membranes. The smallest flexibility is observed in the SM membrane but not in the cholesterol enriched membrane. This correlates with much smaller RMSD and much faster equilibration of the open form of protein in the SM membrane (Figure 2A,B). The drastic decrease of flexibility in SM is more than three times more pronounced than in the case of the closed form of receptor in the regions 220–235 and 252–269 (Figure 2D). Figure 3 visualizes these regions in the aligned crystal structures of the closed and open forms of the receptor.

**Figure 4:**
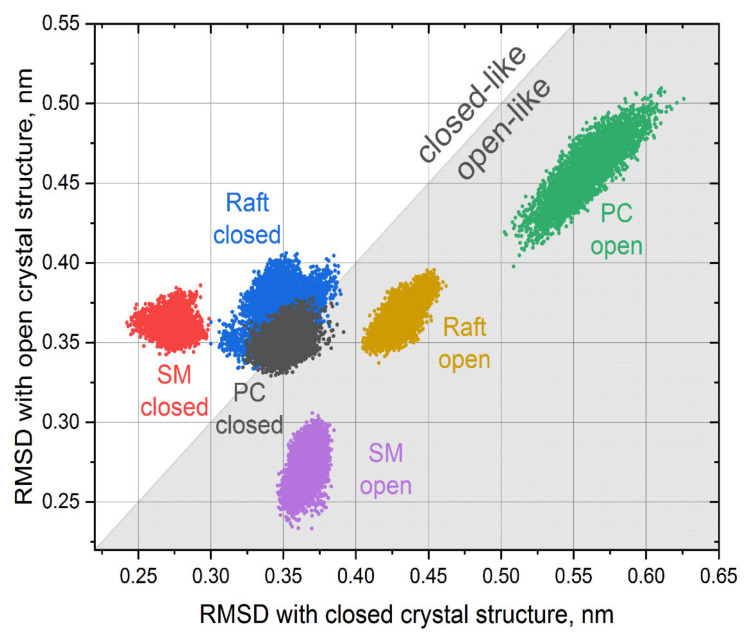
Root mean square deviations (RMSDs) of the TM6 region with closed and open crystal structures of P2Y12. Each point corresponds to a single simulation frame and the clouds of points are colored according to system being simulated. The regions above (white) and below (shaded) the diagonal correspond to the structures with conformations of TM6 resembling one of the closed and open crystal structures, respectively.

We performed an in-depth analysis of the conformational mobility of TM6 by computing the RMSD of the region 252–269 with both closed and open crystal structures of P2Y12 after alignment on the rest of the membrane core of the protein (residues 20–300). Only equilibrated parts of the trajectories were used for analysis. The results are shown in Figure 4.

The conformation of TM6 helix was stable in all simulations experiencing only slight fluctuations, which is obvious from well-localized clouds of points corresponding to each trajectory. In the simulations that started from the closed structure, the orientation of the TM6 helix evolved significantly in the cases of PC and raft environments and stabilized in very similar intermediate conformations between closed and open crystal structure (overlapping gray and blue clouds in Figure 4 are close the diagonal). In the case of the SM environment, the conformation of TM6 remained closer to initial closed structure (red cloud in Figure 4) and is distinct from the other two environments.

The behavior of the TM6 in the simulations, which started from the open structure, differs significantly. Each environment possesses its own unique conformation of the TM6, which are quite different from each other. In the case of the PC environment, the conformation of the MT6 drifted quite far away from both closed and open structures (green cloud in Figure 4). In the cases of raft and SM environments, the deviations from the crystal structures are smaller. It is interesting that both clouds corresponding to SM environments (red and pink) are almost perfectly symmetric in respect to the diagonal, and the TM6 conformation in this environment remains close to initial ones (either closed or open). This suggests that the protein is the most rigid and less prone to conformational changes in the SM environment, which is in agreement with RMSF data in Figure 2.

We did not see any spontaneous transitions between open-like and closed-like conformations of the TM6 in our simulations. However, we observed the multitude of its conformations, which strongly depend on initial conditions and the lipid environment. This allows us to speculate that the TM6 may behave as a flexible “gate”, especially in a less ordered lipid environment, which allows a large conformational mobility of P2Y12. The time scale of these gating motions is likely to be far beyond the length of trajectories in this study and requires extensive dedicated simulations to be detected directly.

We also analyzed the dynamics of the P2Y12 binding pocket directly by means of the principal components analysis. The three top principal components corresponding to the largest eigenvalues were computed for each system. All atoms of the residues contributing to the binding pocket were used after aligning trajectory frames to the average conformation of the P2Y12 transmembrane core. The normalized overlaps between the covariance matrices were computed between different simulated systems (Supplementary Table S2). There is no significant overlap of the top principal components in any pair of simulated systems—all normalized overlaps are in the range 0.2–0.33 (one means that the principal components are identical, while zero means that they are completely different). There is no clear correlation between the principal component overlaps and the fluidity of the membrane environment or initial protein form. Thus, our relatively short simulations do not allow mapping the influence of the membrane environment directly onto the motions of the protein binding pocket. Much longer simulations, which sample characteristic motions of the pocket better, may succeed in this.

It is possible to conclude that the decrease of membrane fluidity and increase of the membrane ordering leads to a general decrease of the protein flexibility. This effect is much more pronounced for the open form of the protein. Particularly, the flexibility of TM6 (residues 252–269), which is responsible for the key structural differences between open and closed states, decreases up to 40% in an SM environment and up to 25% in a raft environment. The flexibility of the intracellular loop region 220–235 decreases up to 70% in the SM environment and up to 60% in the raft environment. This points out the importance of the lipid environment on the flexibility of this protein. It also proves that the models of P2Y12, which do not take into account of their lipid environment, could lead to artifacts.

### 3.2. Docking Results

**Figure 5:**
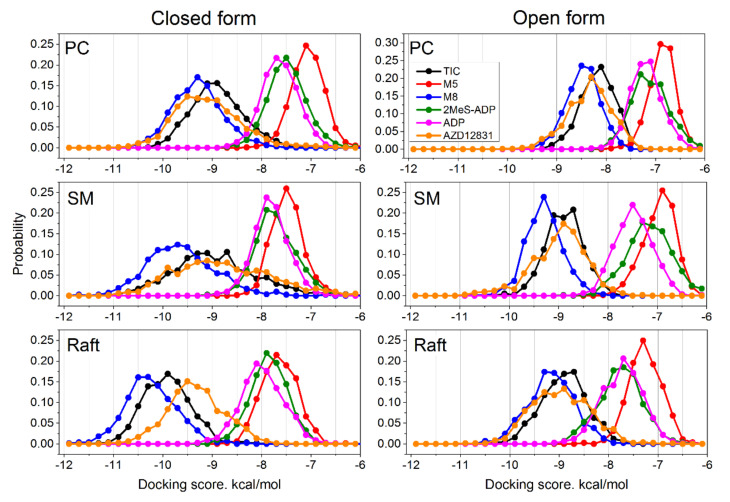
Distributions of the docking scores for studied ligands in different membrane environments.

The ensemble docking utilized in this study allowed us to sample multiple conformations of the protein for each ligand. It takes into account changes of the protein flexibility in each of the studied lipid environments. The distributions of obtained docking scores are shown in Figure 5.

The first remarkable feature is the binding scores of TIC and its metabolites. It is clearly seen that the active metabolite M8 shows stronger binding than pristine TIC (the distribution is shifted to the left) in all membranes and in both protein conformations. The other metabolite M5 shows dramatically worse binding than TIC (the distribution is shifted to the right substantially). The difference between the peaks of TIC and M8 distributions is ≈0.3–0.5 kcal/mol, while the difference between TIC and M5 is ≈2–2.5 kcal/mol in all membranes. This feature corroborates well with the experimental data, which show that the primary metabolite of ticagrelor (M8) is also active with potency equal to the parent compound [12].

The specific agonist 2MeS-ADP binds a bit worse than pristine ADP in most cases; the difference between them varies from zero to ≈0.5 kcal/mol. This difference is systematic and thus unlikely to be an artifact of our docking methodology, but its relevance for the real receptor is not clear. The binding energy of the AZD1283 antagonist varies from one system to the other but remains comparable to or slightly better than that of TIC and much better than that of ADP. One notable exception is the raft membrane in closed form, where AZD1283 binds ≈0.5 kcal/mol weaker than TIC.

**Figure 6:**
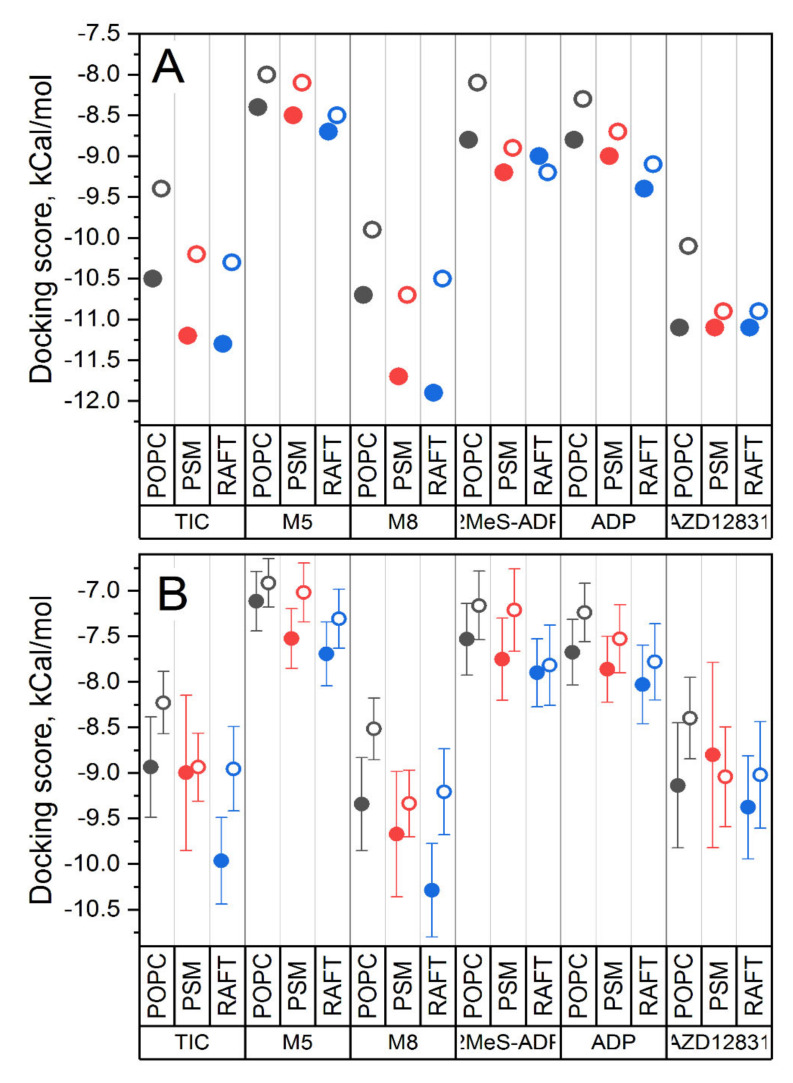
Best and average binding scores for all studied systems. (**A**) The best binding scores. (**B**) The average binding scores with the corresponding standard deviations. The ligands are shown on the bottom and three different membrane environments are shown on top of each ligand. The data points are colored according to membrane environments for clarity (black for PC, red for SM and blue for the raft).

In general, the binding scores of TIC, its active metabolite M8, and AZD1283 are better than those of ADP, which means that these compounds could serve as concurrent antagonists. This correlates well with the experimentally observed action of these compounds. There is a strong and systematic influence of the lipid environment on the ligand binding—the binding of all ligands becomes stronger as the membrane rigidity increases. Figure 6 shows the best and average binding scores for all studied systems. The binding becomes stronger in a raw PC → SM → raft, i.e., with the increase of membrane rigidity.

Another systematic trend concerns the comparison of binding with respect to the two forms of the receptor. In general, the binding of all ligands to the closed form of protein (solid symbols in Figure 6) is stronger in comparison to the open one (open symbols). This effect is especially pronounced for ticagrelor and its metabolites M5 and M8 and the least pronounced for AZD1283. This is in striking contrast to the results of previous docking studies, where the static crystal structures of P2Y12 were used [27]. In these studies, the docking of ticagrelor and similar compounds with bulky N6 substituents to the closed form of protein was not successful. It was suggested that the binding pocket in the closed crystal structure is too small to fit ticagrelor and its homologues because the bulky N6 substituent clashes sterically with inwardly tilted TM6.

This demonstrates that accounting for internal protein dynamics, modulated by its lipid environment, is crucial for correct assessment of the ligand binding to P2Y12.

### 3.3. Docking to The Crystal Structures vs Ensemble Docking

The striking discrepancy between our ensemble docking results and the previous rigid docking studies stimulated us to perform an in-depth comparison between these two techniques. We performed docking to the crystal structures of closed (4PXZ:A) and open (4NTJ:A) forms of P2Y12 using the same setup as for the ensemble docking. The results are shown in Table 2.

The best docking scores for the crystal structures differ dramatically from the results obtained in ensemble docking. In the case of crystal structures, the binding to open form is stronger for all ligands, while for ensemble docking, the trend is strictly opposite, and the binding to closed form is stronger. The pattern of binding scores for different ligands is strikingly different as well for the closed form. Particularly M8 and AZD12831 bind substantially weaker than TIC in the crystal structures. In ensemble docking, the situation is again the opposite: M8 and AZD12831 bind stronger than TIC.

In the case of the open form of protein, the differences between ensemble docking and the docking to crystal structure are less pronounced, and the trends for different ligands are much more consistent.

In the closed form of the protein, the volume of the binding pocket is restricted by the TM6 helix, which is kinked inwards and may impose steric restraints on the ligands. The static crystal structure is especially unfortunate in terms of such restraints. The ligands do not fit well into the pocket and exhibit weak binding. MD simulations allow equilibrating positions of TM6 side chains and to sample different orientations of this helix (see Figure 4), including those that are free from the steric clashes with the ligands. As a result, the ensemble docking reveals favorable docking poses with much better binding scores and demonstrates completely different trends among the ligands.

These observations emphasize the fact that collecting statistics over many protein conformations in ensemble docking allows comparing docking score distributions as a whole, which leads to reliable and unambiguous results. The dynamics of the protein, particularly its flexibility and fluctuations, are affected dramatically by the lipid environment. Such dynamical effect could never be correctly rendered by the docking to single protein conformation. Docking to the single crystal structures of P2Y12 should be avoided because it produces incorrect and misleading results, as it is evident from Table 2.

### 3.4. Comparison of the Binding Sites

Due to the large number of docking simulations, we can estimate the probability of each ligand to be in contact with particular protein residues. The results of this analysis are shown in Supplementary Figure S3.

The pattern of binding probabilities changes with lipid environment and the protein form, but these changes are surprisingly similar for different ligands despite their different chemical nature. Detailed analysis of observed fine differences between the ligands is beyond the scope of this work.

The number of residues with high binding propensity is significantly larger in a PC environment in comparison to SM and raft environments. Particularly, in the closed form of protein in the PC environment, there are two additional groups of residues with high binding probability: 111–115 and 197,201,240,248 respectively. In the open form of protein in the PC environment, there is another additional group: 154,155,157. The raft environment exhibits two residues with high binding propensity, which are absent in other environments: 88 for closed and 18 for open form, respectively.

However, these results should be interpreted with caution because they could be biased by the limited sampling in the particular MD trajectory. Ideally, multiple independent MD and ensemble docking simulations should be performed in each lipid environment, but this requires a prohibitively large amount of time and computational resources.

Comparison with crystallographic structures show that there is a partial match between the residues with highest binding probability in our simulations and the binding pockets in closed and open crystal structures, which correspond to the complexes of P2Y12 with the agonist 2MeS-ADP and the antagonist AZD1283, respectively. In the closed structure, the residues reported to bind with 2MeS-ADP are 19, 93, 97*, 101*, 102, 105,106, 109, 152, 155*, 156, 159, 163, 175*, 179, 187*, 190*, 191, 194*, 256, 259*, 263, and 280* (the matching residues in our docking simulations are marked by asteriscs). In the open structure, the residues reported to bind with AZD1283 are 101*, 102, 105, 106, 109, 152, 155*, 156, 159, 187*, 190*, 191, 194*, 195, 252, 255*, 256, 259*, 260, 276*, 279*, 280*, and 283.

**Figure 7:**
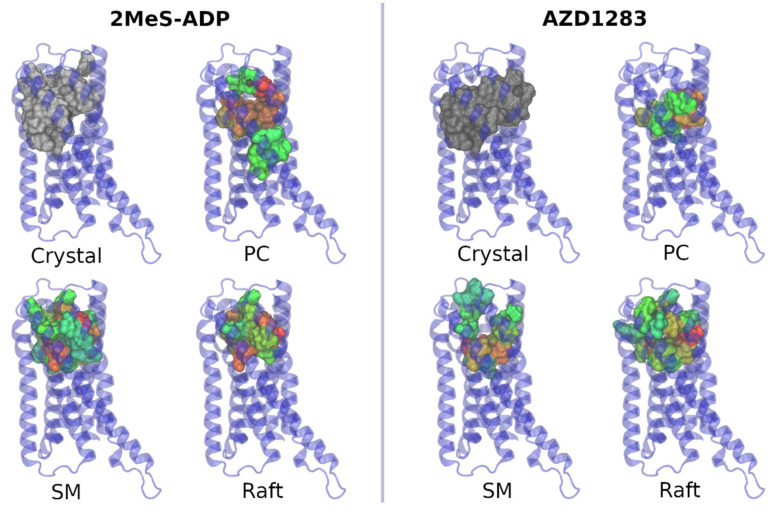
Binding pockets for 2MeS-ADP and AZD1283 in the corresponding crystal. Green corresponds to the smallest probability; red corresponds to the largest. In the case of the crystal structure, the uniform gray color is used.

Spatial locations of the binding pockets for 2MeS-ADP and AZD1283 in our simulations are similar to ones present in the crystal structures, but there is no exact correspondence, as it is evident from Figure 7. Different sets of residues that are involved in ligand binding are observed in each lipid environment.

## 4. Limitations and Perspectives

The major limitation of the current work is limited sampling caused by rather short MD trajectories. These trajectories are sufficient to generate useful ensemble of conformations for docking, which is confirmed by the fact that we managed to dock all ligands successfully to both forms of protein. However, much longer trajectories are needed for comprehensive study of the protein dynamics in the membrane environment. A typical timescale of the physiological conformational changes in the family of GPCR proteins is likely to be in the milliseconds range, which is not accessible in all-atom MD simulations. We presume that in the present study, we mostly see the influence of the hindrance, caused by the lipid environment, on the relaxations of two initial states, which are represented by the closed and open crystal structures. Indeed, the lateral diffusion of the lipids is an order of magnitude slower in SM and raft membranes in comparison to the PC one (Supplementary Table S1), which is likely to dump volumetric relaxations of the protein in these more rigid membranes effectively. Nevertheless, even such limited sampling of the protein dynamics reveals a pronounced effect of the membrane fluidity and ordering on the ligand binding.

Thus, our work provides proof of the principle for the influence of the membrane environment on the ligand-binding propensity of the P2Y12 receptor. Further simulations with larger time scales, multiple replications, and realistic membrane compositions are needed to determine the concrete mechanism of this phenomenon.

## 5. Conclusions

The MD and ensemble docking simulations of the full-length P2Y12 receptor in different membrane environments show that the active ticagrelor metabolite M8 (AR-C124910XX) binds to the receptor stronger than ticagrelor itself, while the metabolite M5 (AR-C133913XX) shows much weaker binding. With the increase of membrane stiffness and ordering, the binding of ticagrelor and both its metabolites becomes stronger with the best binding scores observed in the raft-like sphingomyelin–cholesterol membrane. Our results suggest that this effect is caused by the decrease of the overall protein flexibility and relaxations in the rigid membrane environment rather than due to distinct conformational changes or the rearrangement of its active site. Our data provide first direct evidence of the strong influence of the membrane environment on the ligand binding in P2Y12 receptors and suggest an optimal functioning of this protein in the lipid rafts.

## Figures and Tables

**Table 1:** Lipid and ion content of the studied systems.

System	Lipids	Na^+^	Cl^–^
PC	POPC, 220	20	39
SM	PSM, 220	18	37
Raft	PSM, 112; Cholesterol, 112	21	40

**Table 2:** Best docking scores for the docking to crystal structures of closed and open forms of the protein. The corresponding best docking scores from ensemble docking in the palmitoyl-oleoyl-phosphocholine (POPC) environment are shown for comparison.

	Closed Form		Open Form	
Ligand	Crystal	Ensemble	Crystal	Ensemble
TIC	–6.3	–10.5	–9.2	–9.4
M5	–6.8	–8.4	–7.3	–8.0
M8	–4.7	–10.7	–9.3	–9.9
2MeS-ADP	–4.9	–8.8	–7.3	–8.1
ADP	–7.7	–8.8	–7.6	–8.3
AZD12831	–4.5	–11.1	–9.3	–10.1

## Data Availability

Data are available on demand.

## Supplementary Materials

The following are available online at https://www.mdpi.com/article/10.3390/pharmaceutics13040524/s1, Figure S1: Root mean square deviations of the transmembrane part of P2Y12, Figure S2: Interaction energies of the extracellular (residues 1–19) and intracellular (residues 301–342) P2Y12 termini with the lipid bilayer for different membrane compositions and protein forms. Figure S3: Probabilities of particular residues to be in contact with docked ligands in ensemble docking simulations. Table S1: Lateral diffusion coefficients of the lipids in the studied membranes and Table S2: Normalized overlaps between the covariance matrices computed for top three principal components of the P2Y12 binding pocket.
