# Supplementary material for: Membrane Environment Modulates Ligand-Binding Propensity of P2Y12 Receptor
Source: Pharmaceutics. 2021 Apr 9;13(4):524. doi: 10.3390/pharmaceutics13040524 (PMC8069422; doi:10.3390/pharmaceutics13040524)
Supplement: Supplementary file 1 [file pharmaceutics-13-00524-s001.pdf]

pharmaceutics

Supplementary Materials: Membrane Environment Modulates Ligand Binding Propensity of P2Y12 Receptor

Fatemeh Haghighi, Semen Yesylevskyy, Siamak Davani and Christophe Ramseyer

Citation: Haghighi, F.; Yesylevskyy, S.; Davani, S.; Ramseyer, C. Membrane Environment Modulates Ligand Binding Propensity of P2Y12 Receptor. *Pharmaceutics* **2021**, *13*, 524. https://doi.org/10.3390/pharmaceutics13040524

Academic Editor: Fabrizio Dal Piaz

Received: 9 March 2021
Accepted: 6 April 2021
Published: 9 April 2021

Publisher's Note: MDPI stays neutral with regard to jurisdictional claims in published maps and institutional affiliations.

Copyright: © 2021 by the authors. Licensee MDPI, Basel, Switzerland. This article is an open access article distributed under the terms and conditions of the Creative Commons Attribution (CC BY) license (http://creativecommons.org/licenses/by/4.0/).

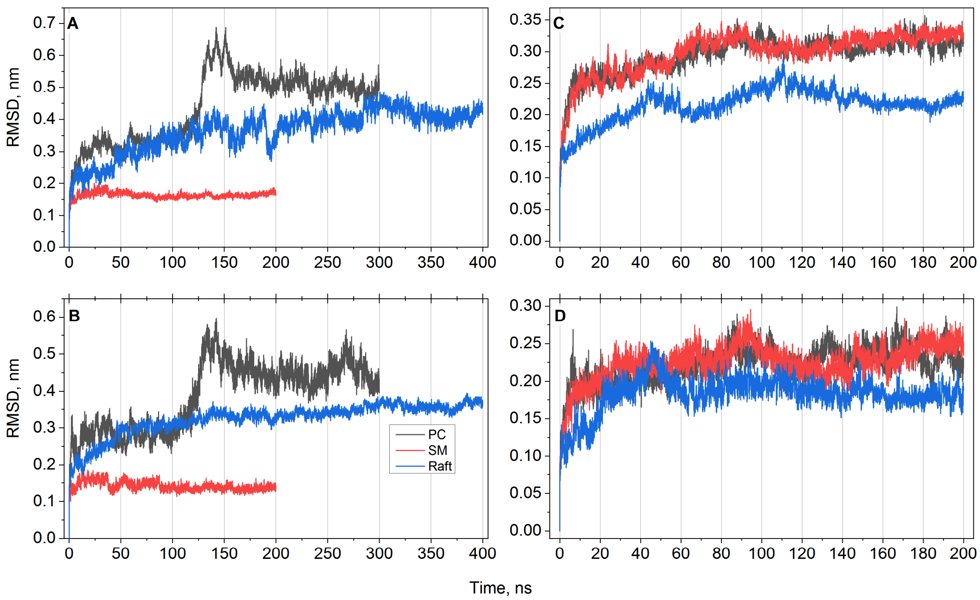

Figure S1. Root mean square deviations of the transmembrane part of P2Y12 (**A**,**C**) and its active site (**B**,**D**) in the course of MD simulations in different lipid environments. **A** and **C** correspond to the open form of the receptor, while **B** and **D** correspond to the closed form.

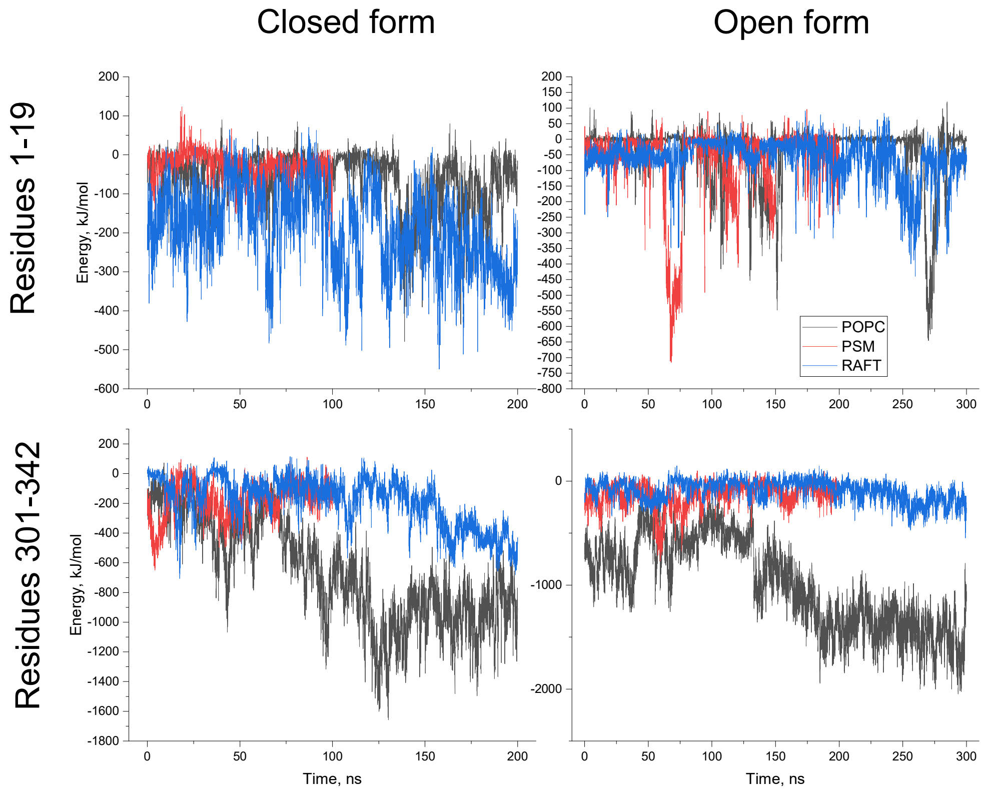

Figure S2. Interaction energies of the extracellular (residues 1–19) and intracellular (residues 301–342) P2Y12 termini with the lipid bilayer for different membrane compositions and protein forms.

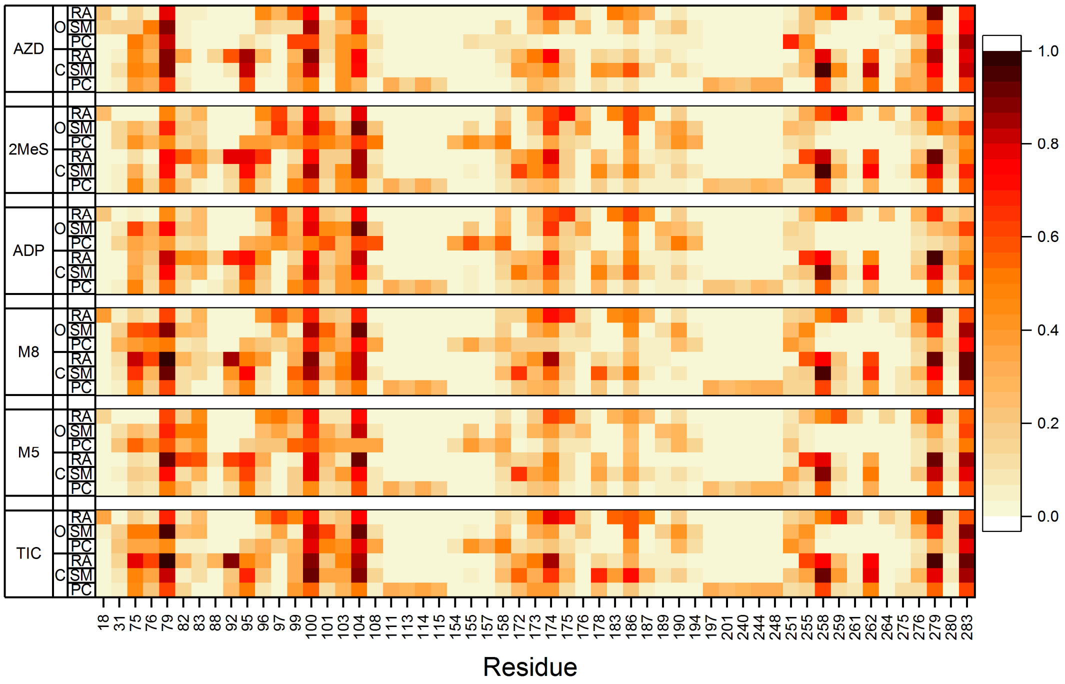

Figure S3. Probabilities of particular residues to be in contact with docked ligands in ensemble docking simulations. Only the residues with the contact probability higher than 25% for at least one ligand are shown. AZD: 6-[4-(benzylsulfonylcarbamoyl)piperidin-1-yl]-5-cyano-2-methylpyridine-3-carboxylate ; 2MeS: 2-Methylthioadenosine diphosphate ; ADP: adenosine diphosphate ; M8: active metabolite AR-C124910XX ; M5: metabolite AR-C133913XX , TIC: ticagrelor ; RA: Raft ; SM: sphingomyelin; PC: phosphatidylcholine

Table S1. Lateral diffusion coefficients of the lipids in the studied membranes.

System	Lipid	D, 10^{-9} cm²/s
PC	POPC	48.0 ± 9.6
SM	PSM	2.3 ± 0.06
Raft	PSM	1.7 ± 0.7
	Cholesterol	1.6 ± 0.9

Table S2. Normalized overlaps between the covariance matrices computed for top three principal components of the P2Y12 binding pocket. * (diagonal of the table) corresponds to the overlap between closed and open forms of the protein simulated in the same membrane environment. ** (top triangle of the table) corresponds to the overlaps between different membrane environments for the closed form of the protein. *** (bottom triangle of the table) corresponds to the overlaps between different membrane environments for the open form of the protein.

	PC	SM	Raft
PC	* 0.27	** 0.29	** 0.33
SM	*** 0.23	* 0.3	** 0.27
Raft	*** 0.33	*** 0.21	* 0.28
